# *Intruder* (DD38E), a recently evolved sibling family of DD34E/*Tc1* transposons in animals

**DOI:** 10.1186/s13100-020-00227-7

**Published:** 2020-12-10

**Authors:** Bo Gao, Wencheng Zong, Csaba Miskey, Numan Ullah, Mohamed Diaby, Cai Chen, Xiaoyan Wang, Zoltán Ivics, Chengyi Song

**Affiliations:** 1 College of Animal Science & Technology, Yangzhou University, 48 Wenhui East Road, Yangzhou, 225009 Jiangsu China; 2 Division of Medical Biotechnology, Paul Ehrlich Institute, 63225 Langen, Germany

**Keywords:** *Intruder*, *Tc1/mariner* transposons, DD38E, Horizontal transfer, Evolution

## Abstract

**Background:**

A family of *Tc1/mariner* transposons with a characteristic DD38E triad of catalytic amino acid residues, named *Intruder* (*IT*), was previously discovered in sturgeon genomes, but their evolutionary landscapes remain largely unknown.

**Results:**

Here, we comprehensively investigated the evolutionary profiles of *ITs*, and evaluated their cut-and-paste activities in cells. *ITs* exhibited a narrow taxonomic distribution pattern in the animal kingdom, with invasions into two invertebrate phyla (Arthropoda and Cnidaria) and three vertebrate lineages (Actinopterygii, Agnatha, and Anura): very similar to that of the DD36E/*IC* family. Some animal orders and species seem to be more hospitable to *Tc1/mariner* transposons, one order of Amphibia and seven Actinopterygian orders are the most common orders with horizontal transfer events and have been invaded by all four families (DD38E/*IT*, DD35E/*TR*, DD36E/*IC* and DD37E/*TRT*) of *Tc1/mariner* transposons, and eight Actinopterygii species were identified as the major hosts of these families. Intact *ITs* have a total length of 1.5–1.7 kb containing a transposase gene flanked by terminal inverted repeats (TIRs). The phylogenetic tree and sequence identity showed that *IT* transposases were most closely related to DD34E/*Tc1*. *ITs* have been involved in multiple events of horizontal transfer in vertebrates and have invaded most lineages recently (< 5 million years ago) based on insertion age analysis. Accordingly, *ITs* presented high average sequence identity (86–95%) across most vertebrate species, suggesting that some are putatively active. *IT*s can transpose in human HeLa cells, and the transposition efficiency of consensus TIRs was higher than that of the TIRs of natural isolates.

**Conclusions:**

We conclude that DD38E/*IT* originated from DD34E/*Tc1* and can be detected in two invertebrate phyla (Arthropoda and Cnidaria), and in three vertebrate lineages (Actinopterygii, Agnatha and Anura). *IT* has experienced multiple HT events in animals, dominated by recent amplifications in most species and has high identity among vertebrate taxa. Our reconstructed *IT* transposon vector designed according to the sequence from the “cat” genome showed high cut-and-paste activity. The data suggest that *IT* has been acquired recently and is active in many species. This study is meaningful for understanding the evolution of the *Tc1/mariner* superfamily members and their hosts.

**Supplementary Information:**

The online version contains supplementary material available at 10.1186/s13100-020-00227-7.

## Introduction

The mobilome is defined as the entire set of mobile (transposable) elements of a genome, which can be categorized into four classes: self-splicing molecular parasites, plasmids, bacteriophages, and transposons [1]. Transposons, which can move about or propagate within the genome, are the major constituents of the mobilome, and are distributed extensively in prokaryotic and eukaryotic genomes [2]. These were once regarded as “junk” DNA, but increasing evidence indicates that they play significant roles in genomic evolution as well as genes [3, 4], and are major determinants of genome size in vertebrates [5, 6]. They can alter the genomic landscape by horizontal transfer (HT) between non-mating species [7–9], and are a key cause of genetic polymorphisms and mutations linked with genomic rearrangements and distinctive characteristics of chromosomes [10], which are increasingly known as major factors in eukaryotic genomic evolution [11, 12]. Transposons can also evolve into new genes by undergoing “molecular domestication”, where the transposons are incorporated into host genes and evolve new functions [13, 14]. In addition, transposons contribute to cis-regulatory DNA components and transcription network modifications [10, 15]. Transposons are typically classified into two classes according to their transposition mode: RNA and DNA transposons, RNA transposons also named as retrotransposons, which need RNA intermediate in their development cycle. While DNA transposons generally do not need RNA intermediate for their transposition, but mostly they form a rather heterogeneous group, composed of cut-and-paste transposons, polintons, and helitrons [2]. Cut-and-paste transposons are the most diverse and abundant category of DNA transposons and comprise at least 17 superfamilies [16]. The best-characterized cut-and-paste DNA transposon is the *Tc1/mariner* superfamily, which was named based on the first *Tc1* (Transposon *Caenorhabditis elegans* number *1*) element identified in *C. elegans* [17] and the first *mariner* element detected from *Drosophila mauritiana* [18]. Most of the *Tc1/mariner* transposon elements vary from 1.3 to 2.4 kb in length and comprise a lone gene encoding a polypeptide surrounded by terminal inverted repeats (TIRs) defining their borders between 5′ and 3′. They seek “TA” sequences to be inserted into the host genome, as a consequence “TA” target site duplications flanking the inserted transposon [19]. At least four families of *Tc1/mariner* transposons, including DD41D/VS, DD37E/*TRT,* DD36E/*IC*, and DD35E/*TR* have been well-described recently [20–23]. While DD34E/*Tc1* [17], DD × D/*pogo* [24], and DD34D/*mariner* [18] were discovered very early and have been reported extensively, and DD × D/*pogo* has been suggested as a separate superfamily of IS630-Tc1-mariner transposon group very recently [25]. Twelve elements (*Tc1*, *Tc3*, *Famar1*, *Minos*, *Mos1*, *Osmar5*, *ISY100*, *Mboumar-9*, *Fot1*, *Impala*, *Thm3*, and *Passport*) of this superfamily are known to be active in their natural form [26–28], and half of them, including *Tc1* [17], *Tc3* [29], *Impala* [30], *Minos* [31], and *Passport* [26], and two artificially reconstructed *Tc1/mariner* transposons, including *Sleeping Beauty* and *Frog Prince* [32], are from the DD34E/*Tc1* family, while DD35E/*TR* [22] and DD36E/*IC* [21] were discovered as new families in our previous studies: they are strongly linked to DD34E/*Tc1* phylogenetically but form distinct sibling clades from DD34E/*Tc1* and seem to have originated from this family. DD36E/*IC* is distributed in both vertebrates and invertebrates, including insects, arachnids, jawless fish, ray-finned fish, frogs, and bats [21], while DD35E/*TR* displays a restricted taxonomic distribution in the animal kingdom, and has only been detected in three classes (ray-finned fish, Anura, and Squamata) and 91 species of vertebrates [22].

Previously, a *Tc1/mariner* transposon family (DD38E), here named *Intruder* (*IT*), was identified in sturgeon (Acipenseridae) genomes [33]; however, the evolutionary landscape of this family, particularly its phylogenetic relationship with other families of the *Tc1/mariner* group, remains largely unknown. Here we describe the evolutionary profile of *IT*, including its distribution, phylogenetic position, structural organization, and HT in eukaryotic organisms; we have also functionally characterized the transpositional activity of a naturally occurring, intact *IT* sequence.

## Results

### DD38E/IT is distributed among invertebrates and vertebrates

**Fig. 1:**
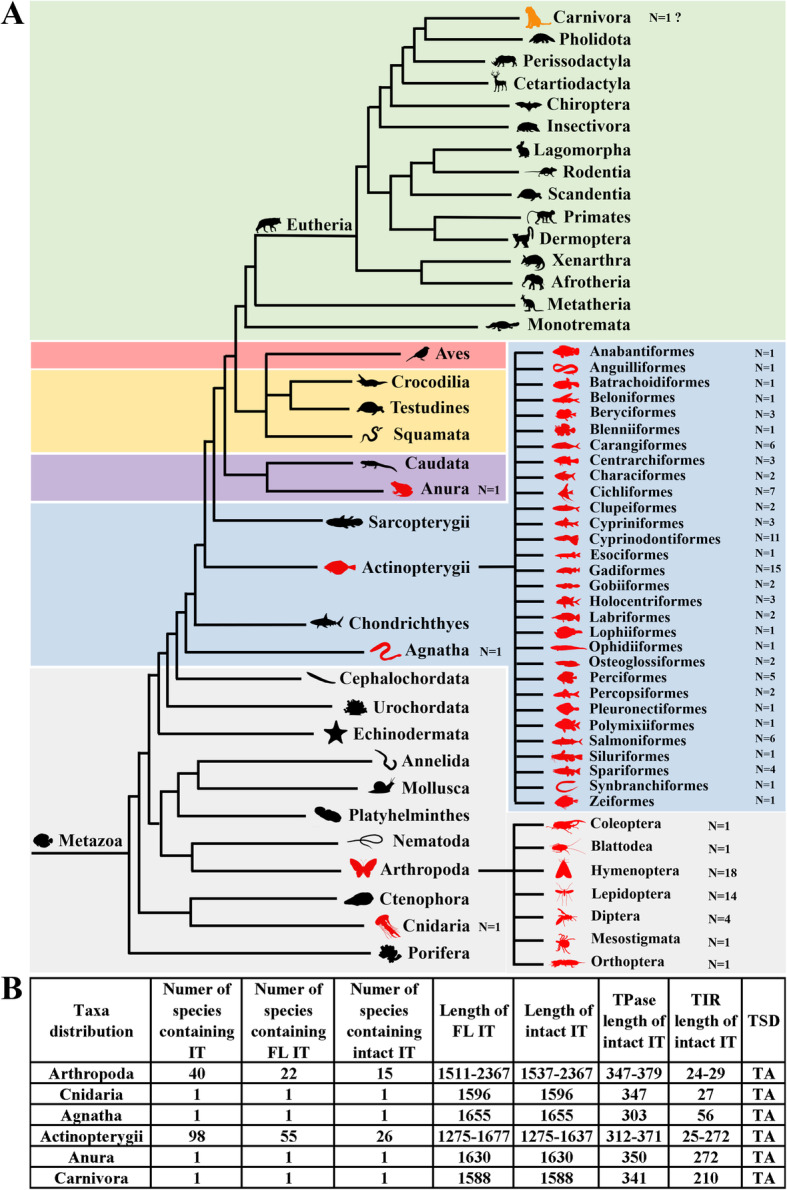
Taxonomic distribution of DD38E/*IT*. **a** Taxonomic distribution of *IT* elements in the animal kingdom. N represents the number of species with *IT*. **b** Description of *IT* elements in six lineages including the number of species with these elements, full length (FL) of the elements, amino acid (aa) numbers of transposases (TPase), lengths of terminal inverse repeats (TIRs) and target site duplications (TSDs)

To assess the distribution of *IT* among species, a TBLASTN search against all the available prokaryotic (archaea and bacteria) and eukaryotic (chromista, plantae, animalia, protozoa, and fungi) genomes placed at the NCBI database was performed using the sturgeon *IT* transposase sequence as the query. This revealed that *IT* has a restricted taxonomic distribution compared with the families of DD41D/VS [23] and DD37E/*TRT* [20] of *Tc1/mariner* transposons, for which the taxonomic breadth has been well defined. Considerable hits encoding the preserved DD38E motifs were identified only in the animalia among eukaryotes, where *ITs* were present in 40 species of Arthropoda and one of the Cnidaria in invertebrates, one Agnathan species, one Anuran species, and 98 species in the Actinopterygii in vertebrates (Fig. 1a, b). Although *IT* elements are found as truncated copies in several species, intact copies of *ITs* with putative HT activity were also detected in many species across multiple lineages, indicating that they might be active. In ray-finned fish (Actinopterygii), *IT* elements were detected in 30 orders, and more than half of the species (55/98) contained *IT* elements flanked with TIRs, designated as full-length *IT*, but in only 26 species do they encode intact transposases, here designated as intact *ITs*. In the phylum Arthropoda, *IT* elements are present in 40 species of seven orders, with 22 species containing full-length *IT* copies, and 15 species harbouring intact *IT* elements. Intact copies of *IT* were also detected in the Anura and Cnidaria, but in the Agnatha, the *IT*s are present as full-length copies but code a truncated transposase (303 amino acids, aa; Fig. 1b and Supplementary Table S1).

In addition, about 20 *IT* copies (> 80% identity and 40% coverage) were also detected in the assembled genome of the domesticated cat (Supplementary Figure S1). However, all these copies were characterized in small contigs (1014–1588 kb) and most were found to be flanked by TA repeat regions (1000–2000 bp; Supplementary Figure S2), indicating possible sequence contamination. DNA samples from the genome of the Abyssinian cat breed and other domesticated breeds were also used to amplify the *IT* copies by PCR, followed by TA cloning and sequencing; however the PCR product bands were not as specific as expected (Supplementary Figure S3), and we did not obtain any positive results by sequencing over 20 clones, which confirmed again that *IT* in the assembled cat genome likely represents sequence contamination. By using BLAST at NCBI database to search against the nucleotide collection (nr/nt), we found these *IT* copies display very high sequence similarity to Tc1 transposons identified in the sturgeon genome (Supplementary Figure S4), indicating that this might be the source of contamination.

### Similar distribution patterns of IT and IC transposon sequences

**Fig. 2:**
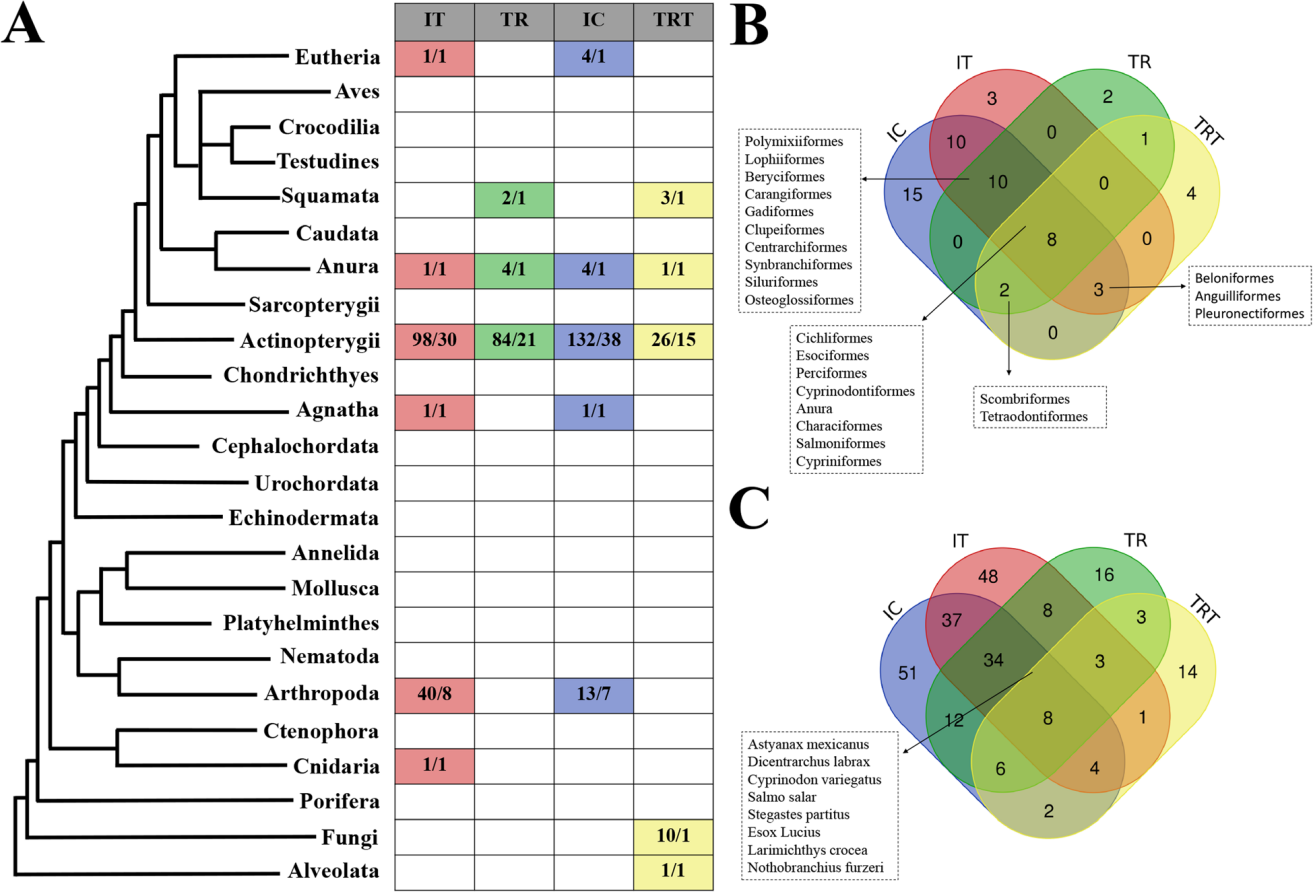
Distribution patterns of DD38E/*IT*, DD35E/*TR*, DD36E/*IC* and DD37E/*TRT*. **a** Distribution of *IT*, *TR*, *IC* and *TRT* transposons. The numbers of species/orders detected for each family are indicated for each lineage. **b**, **c** Venn diagrams of distribution patterns across orders and species. Figures were generated from the Supplementary File Text S3

Comparison across the *IT*, *TR*, *IC* and *TRT* sequence families revealed that *IT* and *IC* display a very similar taxonomic distribution pattern (Fig. 2a). Over 90% of orders with *IT* detected (31/34) overlap with the orders with *IC* detected; only three are *IT*-specific, and 58% of the species with *IT* detected (83/143) overlap with the species with *IC*. Both *TR* and *TRT* elements were found to be distributed in the Anura, Squamata, and Actinopterygii, but the similarity was relatively low at order and species levels compared with that between *IT* and *IC*, with about 30% of orders with *TR* detected (11/33) overlapping with the orders with *TRT*, or about 61% (11/18) overlapping with these of *TR* (Fig. 2a–c). On the other hand, the taxonomic distributions of *Tc1/mariner* families (*IT*, *TR*, *IC*, and *TRT*) in animals also revealed that they share some common hosts. Thus, some orders and species seem to be more hospitable to *Tc1/mariner* transposons than others, which has also been noted in a recent study [23]. The Actinopterygii and Anura tend to be more susceptible to the invasions of *Tc1/mariner* transposons, as all well-defined close sibling families of DD34E/*Tc1* (*IT*, *TR*, *IC* and *TRT*) were detected in these lineages. One order of Amphibia (Anura) and seven Actinopterygian orders (Characiformes, Cichliformes, Cypriniformes, Cyprinodontiformes, Esociformes, Perciformes and Salmoniformes) are the most common orders with HT events and have been invaded by all four families (*IT*, *TR*, *IC* and *TRT*) of *Tc1/mariner* transposons (Fig. 2b). Fifteen other orders are also very common reservoirs of *Tc1/mariner* transposons and have been invaded by at least three families. Eight species in the Actinopterygii (*Astyanax mexicanus*, *Cyprinodon variegatus*, *Dicentrarchus labrax*, *Esox Lucius*, *Larimichthys crocea*, *Nothobranchius furzeri*, *Salmo salar*, and *Stegastes partitus*) were identified as the hosts of most HT events and have been invaded by all four families (*IT*, *TR*, *IC* and *TRT*) of *Tc1/mariner* transposons (Fig. 2c).

### DD38E/IT might originate from DD34E/Tc1

**Fig. 3:**
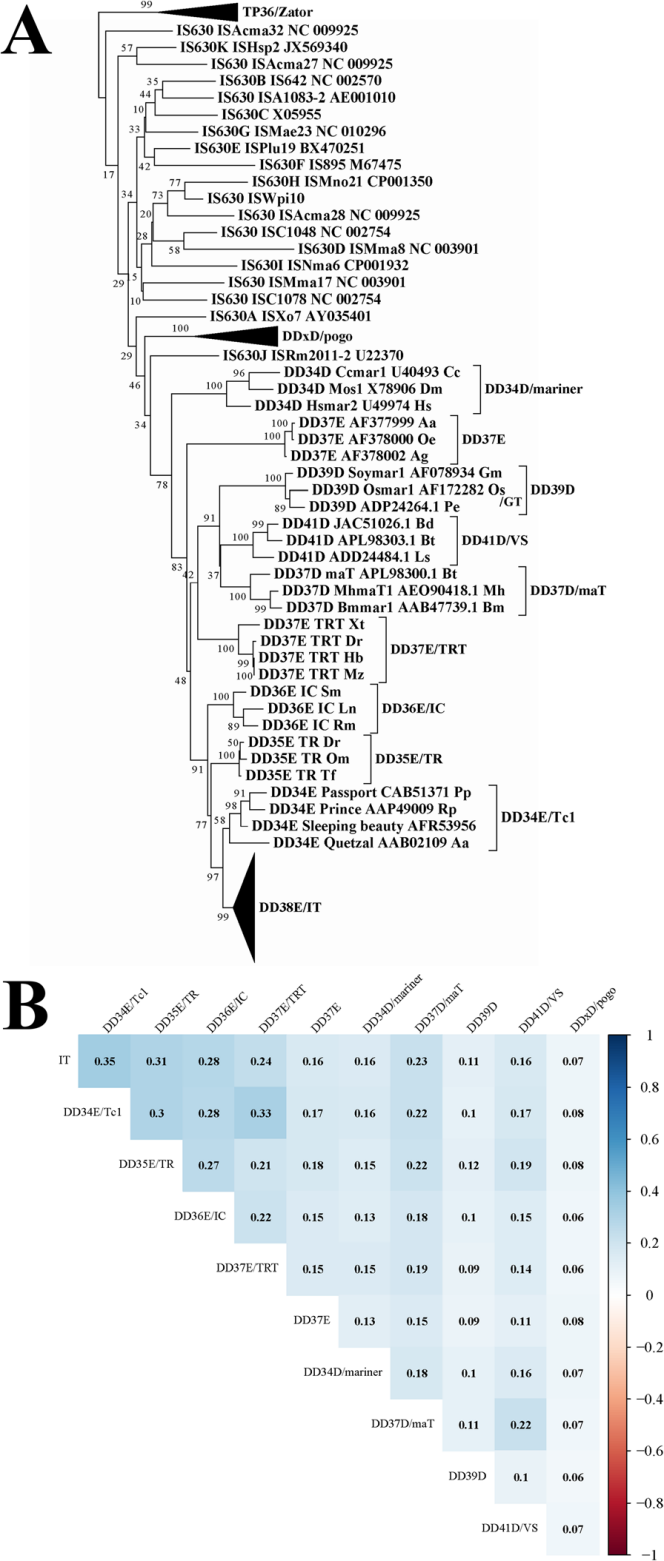
Phylogenetic position of the *IT* family. **a** This phylogenetic tree was generated based on DDE domains by using the Maximum Likelihood method in the IQ-TREE program (http://iqtree.cibiv.univie.ac.at) with an ultrafast bootstrap approach (1000 replicates). The reference families and elements included DD34E/*Tc1*, DD35E/*TR*, DD36E/*IC*, DD37E/*TRT*, DD34D/*mariner*, DD37D/*maT*, DD39D, DD41D/VS, DD × D/*pogo* and *IS630* transposases. *TP36/Zator* was used as an outgroup. **b** Sequence identity matrix of *Tc1/mariner* families by pairwise comparisons among FL transposases

**Fig. 4:**
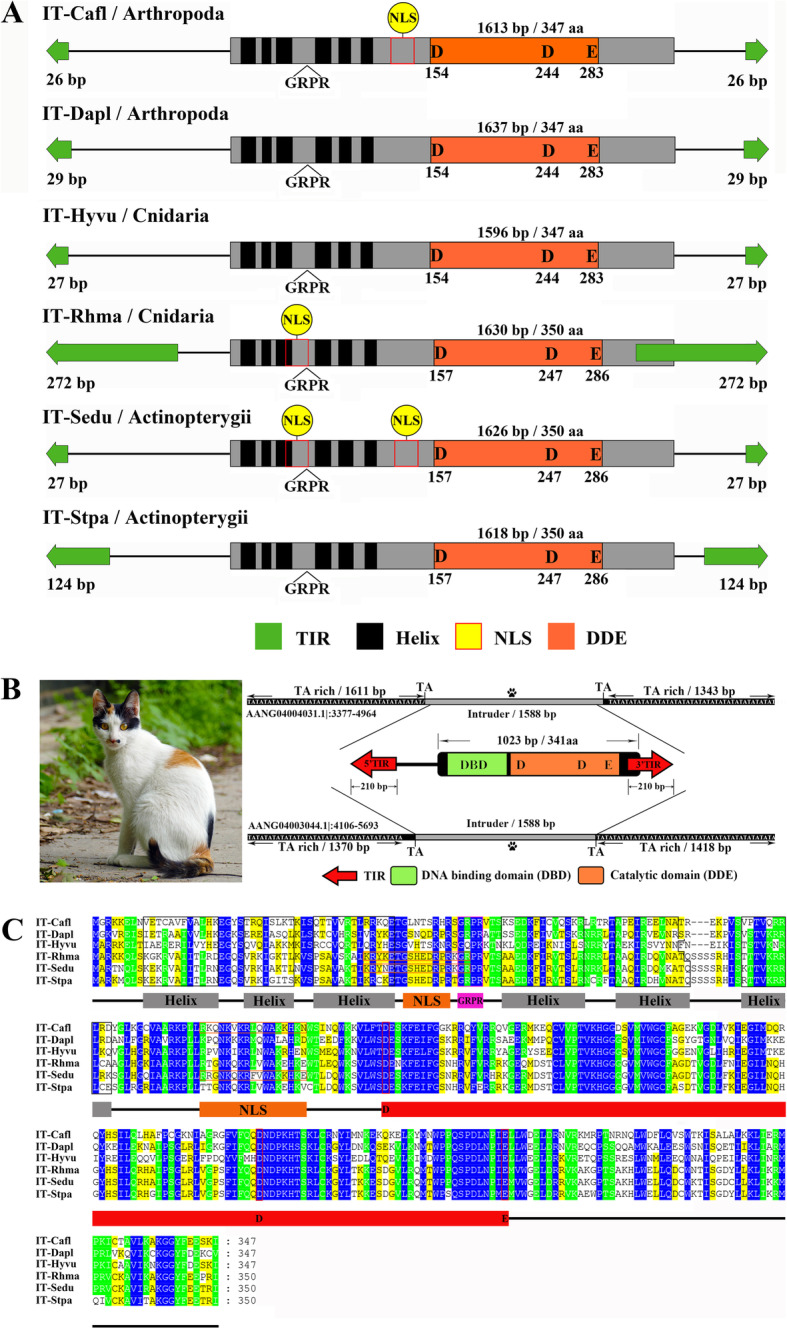
Structural schematic of *IT* transposons. **a** Structural organization of *IT* elements. The green arrows represent TIRs, the black rectangles represent HTH motifs, the black triangles represent GRPR sequences, the yellow circle represents the NLS, the orange rectangles represent catalytic domains, and the grey regions represent transposases. The dotted box represents the portion of the transposases that might be deleted in a particular species. **b** *IT* in the “cat” genome. The grey areas at the top and bottom represent *IT*. We selected copies 1 and 2 to mark the locations in this genome. In the middle is a schematic diagram of the complete *IT* structure. The red arrows represent TIRs, the green rectangle represents the DNA-binding domain, and the orange rectangle represents the catalytic domain. **c** Motifs prediction for IT transposases. This analysis was performed using multiple alignment with Bioedit and with modifications in Genedoc. Species abbreviations: Cafl, *Camponotus floridanus*; *Danaus plexippus plexippus*; Hyvu, *Hydra vulgaris*; Rhma, *Rhinella marina*; Sedu, *Seriola dumerili*; Stpa, *Stegastes partitus*

The phylogenetic trees generated from the alignments of the DDE domains proved that all identified elements belong to the DD38E/*IT* family, which was more intimately linked to the DD34E/*Tc1* and DD35E/*TR*, DD36E/*IC* and DD37E/*TRT* (Fig. 3a and Supplementary Figure S5). The sequence identity matrix also showed that DD38E/*IT* transposases were more intimately linked to DD34E/*Tc1* than other families by an average percentage identity of 35% (Fig. 3b), indicating that DD38E*/IT* could have derived from DD34E*/Tc1*. Most intact *IT* transposons have a total length of 1.5–1.7 kb and contain a single ORF encoding a transposase of 341–379 aa, flanked by two short (< 100 bp) or long (100–272 bp) TIRs (Figs. 1b, 4a, and Supplementary Table S1). Intact *IT* in the “cat” genome, representing the typical structural organization of this family, has a length of 1588 bp, harbouring an ORF coding for a 341 aa transposase, flanked by 210 bp right and left TIRs. Several conserved sequences, including six helix-turn-helix (HTH) motifs, GRPR motifs in the DNA-binding domain, and NLS motifs, which are distinctive of *Tc1/mariner* transposases [19], were determined in the *IT* transposases by in silico forecast, and the DBD domain and DDE signature and its distances in the DDE domain appeared to be strongly conserved throughout the *IT* family (Fig. 4b, c, and Supplementary Figure S6).

### Evidence of multiple HT events of IT transposons in vertebrates

**Fig. 5:**
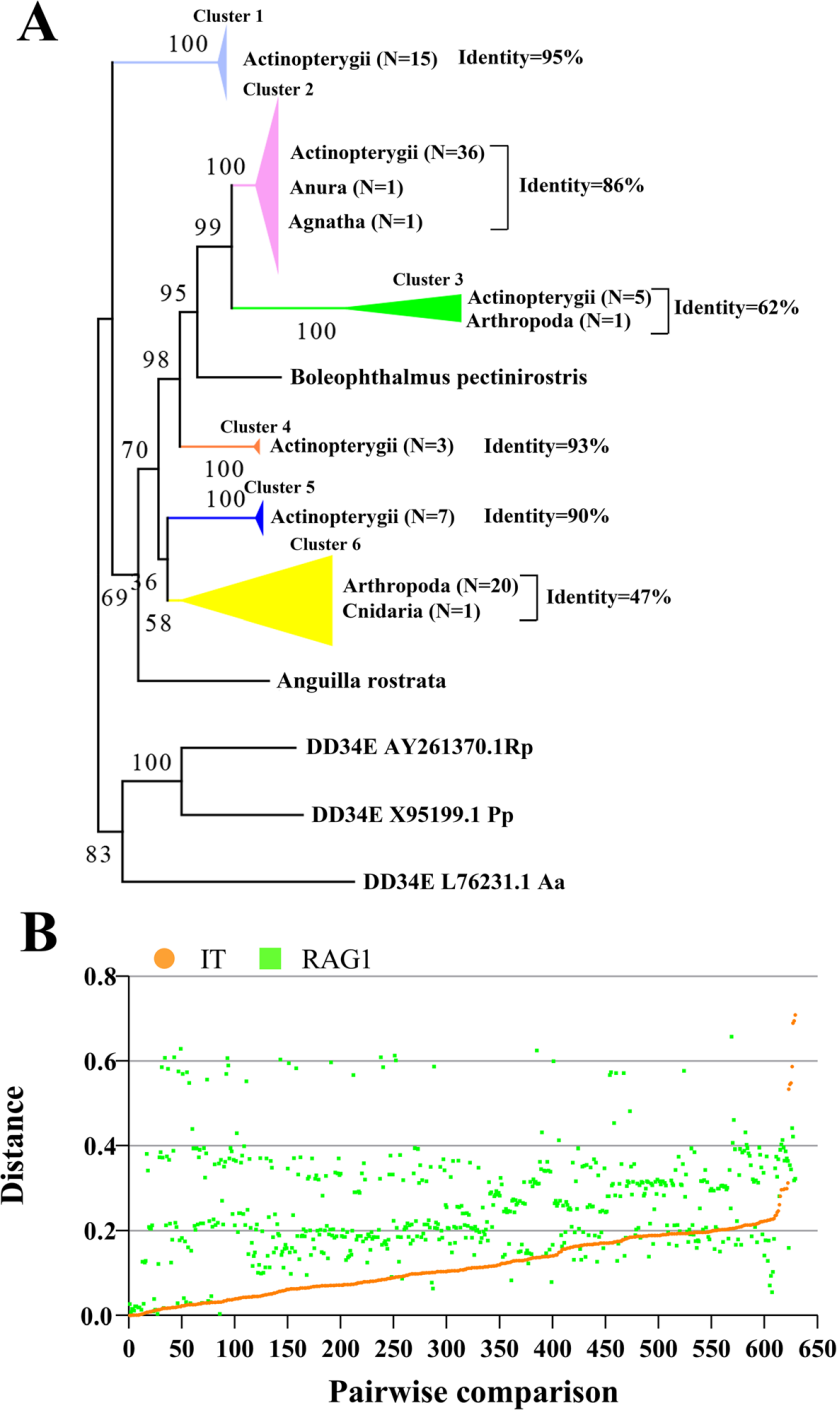
HT analysis of *IT* transposons. **a** Phylogenetic tree based on alignment of the nucleotide sequence of *IT* transposons. The phylogenetic tree was inferred using the maximum likelihood method with the IQ-TREE program (http://iqtree.cibiv.univie.ac.at), and the DD34E/*Tc1* family was used as the outgroup, the identity calculation of each cluster was done using MEGA7. Only consensus or representative sequences were used in this analysis. **b** Horizontal transfer of *IT* transposons. The distance was obtained from all possible pairwise comparisons (*n* = 629; marked on the x-axis) between the 35 (cluster 2), five (cluster 3), three (cluster 4) and seven (cluster 5) species in which *IT* motifs were identified and complete. The coding sequence (CDS) of the *RAG1* gene from the NCBI database is available in Supplementary Table S2

*IT* elements were further classified into six major clusters based on the alignment of the full-length transposon nucleotide sequences: vertebrate species mainly distributed in five clusters (1–5), Clusters 1, 4 and 5 were detected in 15, 3 and 7 species of ray-finned fish, respectively; cluster 2 was identified in one species of jawless fish, 36 species of ray-finned fish, and one species of Anura; cluster 3 was present in five species of ray-finned fish and one species of Arthropoda; while cluster 6—most common in invertebrate species—was present in one species of Cnidaria and 20 species of Arthropoda (Fig. 5a and Supplementary Figure S7). The overall topology of this phylogenetic tree was quite distinct from the established phylogeny of these species from which it is extracted, this may mean the exposure of IT elements to several HT events. To test this assumption, pairwise distances among all consensus sequences or representative sequences of *IT* transposons and *RAG1* coding sequences were calculated. Indeed, for most pairwise comparisons (561/629), the distances measured for *IT* (mean 0.121; SD ± 0.09; range 0–0.709) are far smaller than those measured for *RAG1* (mean 0.259; SD ± 0.12; range 0.04–0.658) (Fig. 5b and Supplementary Table S2), which are typically used to predict HT incidents of transposons in vertebrates [34]. Meanwhile, most of species included in these *IT* pairwise distances engaged a last common ancestor more than 166 million years ago (Supplementary Table S2). Together, these results clearly indicate that the existence of *IT* in several of the main vertebrate lineages tested here results from HT incidents that occurred after these lineages diverged from each other.

### Very recent invasions of ITs in vertebrates

**Fig. 6:**
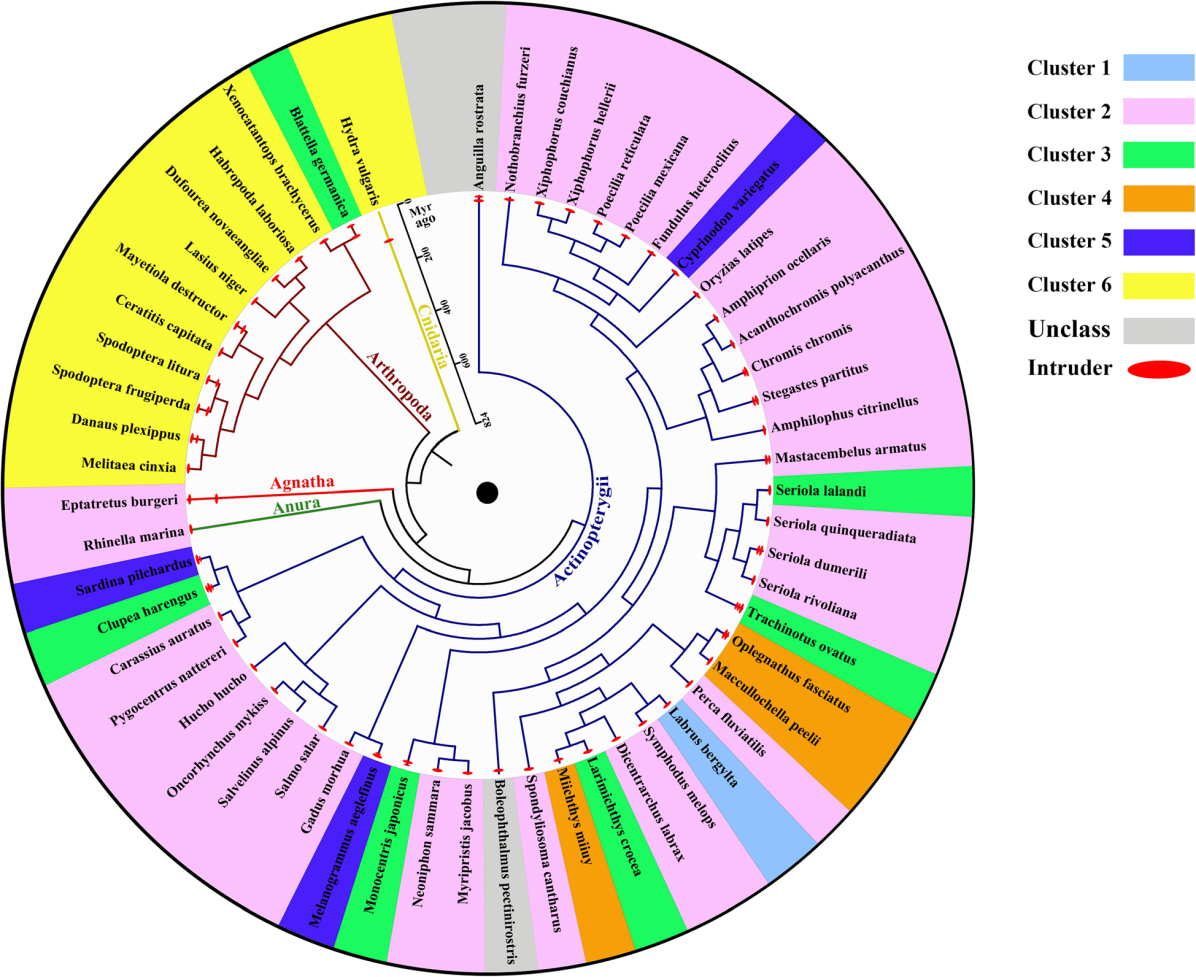
Insertion ages of *ITs*. This taxonomic tree represents the distribution of the species identified in the animal kingdom, and each colour represents a phylogenetic tree cluster. Insert age analysis was calculated by using the RepeatMasker program. The phylogenetic relationships were taken from the TimeTree database

To investigate the evolutionary dynamics of *ITs* in animals, we also compared the insertion ages and sequence identities of *ITs* in these species across clusters, which revealed differential evolutionary dynamics. Most species have experienced recent and sharp peak activities (less than 5 million years ago) of *IT*s, suggesting recent invasions. Some species, such as *Myaetiola destructor*, *Spodoptera litura*, *Spodoptera frugiperda*, *Danaus plexippus* among invertebrates, and *Eptatretus burger*, *Sardina pilchardus*, *Clupea harengus*, *Melanogrammus aeglefinus*, *Oplegnathus fasciatus*, *Trachinotus ovatus*, *Seriola dumerili*, *Mastacembelus armatus* and *Stegastes partitus* among vertebrates experienced multiple waves (two or three) of invasions, whereas more than half of all species, such as *Rhinella marina*, *Hucho hucho*, *Oncorhynchus mykiss* and *Salmo salar*, experienced a single wave of amplification (Fig. 6 and Supplementary Figure S8). In addition, the overall mean sequence identity (50.66 ± 22.21%) of *IT*s across species is similar to that for DD36E/*IC* (52.48 ± 19.19%), but lower than for DD35E/*TR* (82.33 ± 10.01%; Supplementary Figure S9A), but most species in clusters 1, 2, 4 and 5 in vertebrates display very high sequence identities, ranging from 86 to 95% (Fig. 5a and Supplementary Figure S9B–E), indicating very recent HT events of *IT*s in these species. The discovery of intact *IT*s in multiple lineages of animals and high sequence identities in vertebrates, combining recent and sharp peak activities in most animal lineages, suggest that this family is a recently evolved clade of *Tc1/mariner* transposons that might still be active in some of these lineages.

### IT is transpositionally active in mammalian cells

**Fig. 7:**
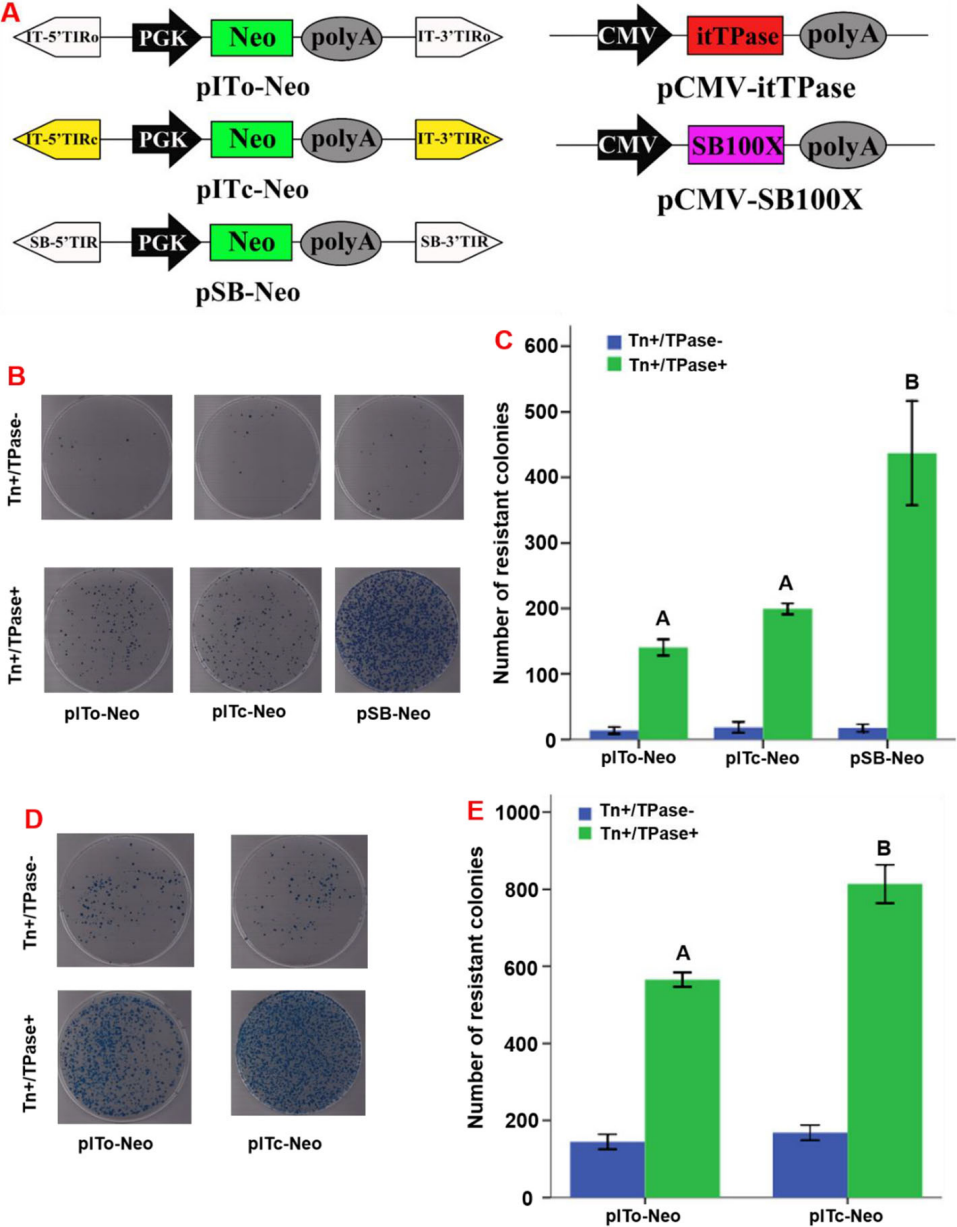
*IT* is transpositionally active in mammalian cells. **a** *IT* and *Sleeping Beauty* (*SB*) transposon vectors for in vitro transposition activity assays. *SB* was used as a positive control. The three TIR vectors—p*IT*o-Neo, p*IT*c-Neo and pSB-Neo—have the same vector frame, and the TIR elements on both sides are the only differences. p*IT*o-Neo is composed of two original TIR sequences of *IT*, p*IT*c-Neo is composed of one consistent TIR and the other original TIR of *IT*, and pSB-Neo is composed of two *SB* TIRs. The two transposase vectors pCMV-itTPase and pCMV-SB100X also have the same frame. **b**–**e** HeLa cells were stably transfected with 1 μg of mixed plasmids (donor and helper plasmids at a 1:1 ratio. For selection, the transfected cells were reseeded onto 10-cm dishes (**b** and **c**, 1:100 plating; **d** and **e**, 1:10 plating). Selection was performed using 1000 mg/ml G418 for 14 days in DMEM. To determine the relative rates of transgenesis, foci of neomycin-resistant cell colonies that remained on each dish were counted after being fixed in 4% paraformaldehyde and stained with methylene blue. Bars represent the mean neomycin-resistant colonies ± standard deviations from three independent experiments

The dual plasmid-based assay [35, 36] was applied to test the transpositional activity of the particular *IT* element identified in the “cat” genome assembly. The donor plasmids, harbouring a PGK promoter and a neomycin phosphotransferase cassette flanked by TIRs can confer G418 resistance in mammalian cells upon transposition into chromosomes. The helper plasmid has an expression cassette of transposases driven by a CMV promoter. The original 210 bp 5′TIRo and 3′TIRo and 1023 bp transposase (341 aa) of the intact copy of *IT* in “cat” genome (AANG04004031.1|:3377–4964), and the consensus sequences of *IT* TIRs (5′TIRc and 3′TIRc), which are 97.62 and 100% identical to the 5′TIRo and 3′TIRo (Supplementary Figure S10), were used to construct vectors. The *SB* transposon together with the SB100X hyperactive transposase [35, 37] was applied as a positive control. A schematic of the donor and helper plasmids is shown in Fig. 7a. The donor plasmid was then mixed with the helper plasmid 1:1 and co-transfected into HeLa cells with subsequent G418 selection. We found that both the *IT* and *SB* transposases displayed substantial transposition activity in human cells (Fig. 7b). The frequency of *IT* transposition was about 45% of *SB* in HeLa cells by measuring the numbers of neomycin-resistant colonies (Fig. 7c). In addition, there were more colonies in the cell group transfected with pITc-Neo plus transposase than with pITo-Neo plus transposase (1% replating selection), indicating that the transposition activity of consensus TIR sequences of *IT* may be higher than that of the original TIR, which was confirmed by using 10% replating selection, where we found a significant difference (*P* < 0.01) between pITc-Neo and pITo-Neo groups (Fig. 7d, e). The integration sites of *IT* in the human genome were recovered by ligation-mediated PCR as described in the methodology and confirmed by Sanger sequencing. These data indicate that *IT* sequences are still functionally active and can potentially undergo HT in mammalian cells.

## Discussion

### Different evolutionary profiles across the Tc1/mariner families

Although diverse *Tc1/mariner* transposons families have been discovered and known for decades, the evolutionary landscapes of some of them are not well characterized, and knowledge of their taxonomic distribution, intra-family diversity and evolutionary dynamics are still fragmented because of the limited historical availability of genome information, such as for DD34D/*mariner*, DD34E/*Tc1*, DD × D/*pogo*, DD37D/*maT*, and DD39D [19, 38]. With increases in genome sequencing data, the whole evolutionary profiles of these DNA transposons can now be well defined and more informative data are available, such as for DD37E/*TRT* [20], DD36E/*IC* [21], DD35E/*TR* [22], DD41D/VS [23] and DD38E/*IT* reported here. However, we still found that *Tc1/mariner* transposons have experienced significantly different evolutionary profiles across families in terms of the complexity of intra-family structure, taxonomic breadth, and evolutionary dynamics. Evidence of invasion by DD34D/*mariner* in fungi [39], plants and animals [40–44]; of DD34E/*Tc1* in protozoans [45], plants [46], and animals [30, 32, 47–53]; of DD × D/*pogo* in protozoans [45], fungi [54–56], and animals [24, 34, 57, 58] support the idea that these families are distributed widely in nature. DD37E/*TRT* transposons also occur widely in eukaryotes and are present in protozoans, fungi and animals [20]. By contrast, the DD36E/*IC* [21], DD35E/*TR* [22], DD41D/VS [23], and DD38E/*IT* families exhibit relatively narrow distributions and are mainly restricted to the animal kingdom, and DD35E/*TR* is only detected in vertebrates [22]. In addition, analyses of evolutionary dynamics have suggested that DD37E/*TRT* [20], DD36E/*IC* [21], DD35E/*TR* [22], and DD38E/*IT* seem to be recently evolved families, and more active and intact copies of these families can be detected in many species across multiple lineages. The evolutionary dynamics and complexities of the intra-family structures of DD34D/*mariner* and DD34E/*Tc1* transposons are still poorly understood, although multiple distinct clades of *mariner* [28] and *Tc1* [59] have been noted.

### Intra-family diversity of DD34E/Tc1

DD34E/*Tc1* is a classical family of *Tc1/mariner* transposons, first found in *C. elegans* in the 1980s [17], and displays extensive distribution in nature [28]. Diverse DD34E/*Tc1* transposons were discovered in multiple lineages of animals, including *Topi* and *Quetzal* in mosquitoes [48, 60], *Impala* in fungi [30], *Minos*, *S* elements and *Bari-3* in fruit flies [49, 50, 53], *Frog Prince* in frogs [32], and *Passport* in fish [26]. The DD34E/*Tc1* group was then divided into at least five distinct clusters based on the DD34E/*Tc1* elements identified from six teleost species in our earlier study, combined with previously reported elements of this family from other laboratories [58]; however, the intra-family diversity is still ambiguous. In addition, one distinct family (*Gambol*) also has the DD34E domain, but is far away from the DD34E/*Tc1* sequence in phylogenetic position, and is close to the DD35E/*IS630* family [61]. Recently, two new families (DD35E/*TR* and DD36E/*IC*) were discovered, and are close to *Tc1* in phylogenetic position [21]. Here, we identified a third new family (DD38E/*IT*) with a varying DDE domain (DD38E), but still retaining a close polygenic relationship with DD34E/*Tc1*. Generally, these three families (DD35E/*TR*, DD36E/*IC*, and DD38E/*IT*) display similar evolutionary profiles with restricted distribution, relatively recent invasion history in most species, and high sequence identity across species [21]. They seem to have originated from the *Tc1* family and have evolved into new clades with varied DDE domains. Thus, these groups might represent subfamilies of *Tc1*, not sperate families of *Tc1/mariner* transposons. Such results demonstrate that the *Tc1* family has high diversity. Systematically characterizing the taxonomic distribution of homologous *Tc1* elements and defining the whole evolutionary landscape of this family will be very interesting and might help to illustrate the family structure of *Tc1*.

### Major reservoir hosts of Tc1/mariner transposons

*Tc1/mariner* families, with well-defined evolutionary profiles, including the DD35E/*TR*, DD36E/*IC* and DD37E/*TRT* families discovered recently, and the DD38E/*IT* family reported here, repeatedly invaded the arthropod phylum of invertebrates and/or the class Actinopterygii of vertebrates, suggested that arthropods and ray-finned fish might be *Tc1/mariner* transposons major reservoir hosts. Furthermore, the evolutionary landscape of transposable elements (TEs) in vertebrates revealed that almost all main kinds of eukaryotic TEs exist in ray-finned fish, which display the highest TE diversity across vertebrate groups [5, 62]. The evolutionary profile of TEs in arthropods also demonstrated that almost all known TEs have been identified in this phylum. This could be explained either because these lineages might be more prone to the exchange of genetic material or are more congenial to them than others, or because they comprise a great diversity of species. Previous reports suggested that some taxa, such as the bats (Chiroptera) are more prone to the invasion of DNA transposons than other types and have experienced multiple invasion events of the main DNA transposon superfamilies (*hAT*, *piggyBac*, *Tc1/mariner* and *helitron*). Bats are also suggested as the main reservoir hosts of many high-impact viruses that cause severe human diseases [63]. By contrast, arthropods have been suggested as major reservoir hosts for many of the negative-sense RNA viruses [64]. These results suggest that some lineages are more susceptible to the invasion of genetic materials than others, although the mechanisms remain largely unknown. Additionally, and in numerical terms, ray-finned fish (Actinopterygii) are the dominant class of vertebrates, comprising half of all living vertebrate species and approximately 32,000 species are recognized within this class [65]; while the phylum of arthropods accounts for more than 80% of all recognized animal species, with values of the number of arthropod species being 5–10 million [66]. Both ray-finned fish (Actinopterygii) and Arthropod lineages have great species diversity, so might serve as the major reservoir of most eukaryotic TEs. On the other hand, it is often believed that TEs facilitates diversification or biological and genomic distinction between organisms [67, 68], TE activity is positively corresponding to the speciation rate in mammals [69] and lineages harbouring recently acquired TE families are also linked with latest speciations [70], suggesting that TE activity might play roles in the facilitation of reproductive isolation, and ultimately in speciation.

### High activity of Tc1/mariner transposons in ray-finned fish

TEs display drastically different evolutionary dynamics across vertebrate groups; thus, recently active DNA transposons are more frequent in ray-finned fish genomes than in birds or mammals [6, 62, 71]. Although multiple mammalian lineages (galagos, murine rodents, opossums, tenrecs, bats and primates) have been invaded by DNA transposons, most of them appear as truncated copies in these genomes, and have lost transpositional activity, except for *piggyBac* domains in bats, which have been reported as being functionally active copies [72]. Assesses of the evolutionary dynamics of DD35E/*TR*, DD36E/*IC*, and DD37E/*TRT* also indicate that many *Tc1/mariner* transposons in ray-finned fish species are recently acquired elements with intact copies and tend to be functionally active. Here, we reconfirmed that the *IT* motifs seem to be highly active in the ray-finned fish lineage with many species containing recent *IT* insertions. In addition, two active transposons in their native form, including *Passport* from flatfish [26] and *Thm3* from silver carp [27], were also identified from the class of ray-finned fish. Here, we have shown that a third *Tc1/mariner* transposon (DD38E/*IT*) encodes functional components required for cut-and-paste transposition in human cells. *IT* displayed a rate of transpositional activity corresponding to up to half the level we observed for the highly active *SB* transposon [37]. Thus, these data reinforce that active *Tc1/mariner* transposons in ray-finned fish are common, and these active TEs might have played key roles in driving the genomic evolution of this group and in their speciation.

## Materials and methods

### Identification of IT transposons

The sturgeon *IT* transposase sequences [33] were employed as a query to investigate genomes available for organisms, including eukaryotes and prokaryotes at the National Center for Biotechnology Information (NCBI) by using the cut-off value of TBLASTN of 1e^− 100^. Alongside 2 kb of flanking sequences, the top uncorrelated hits were retrieved and then used against the host genome to BLAST. All hits with more than 80% identity and 40% coverage were downloaded and aligned to define transposon boundaries using the MAFFT software; the TIRs of *IT* sequences with low genome copies were determined manually. The consensus sequences of *IT* transposons were reconstructed using multiple alignments of *IT* copies from every genome. The new sequences identified were then used as queries to recognize more *IT* elements. In addition, the flanking regions of all *ITs* with very low copy numbers located on short contigs in the genome were checked to ensure they were not sequence contaminations. The copy number of *IT*s in each genome was estimated by using BLAST (40% coverage and 80% identity) with the consensus sequences or representative sequences of *IT*s.

### Sequence analysis and phylogenetic inference

Predictions of the secondary structure of proteins were created using the software PSIPRED (http://bioinf.cs.ucl.ac.uk/psipred/). Putative nuclear localization signal (NLS) motifs were predicted using WOLFPSORT (https://wolfpsort.hgc.jp/). Multiple alignments of full transposases and DDE domains were performed using the MAFFT program [73]. The DDE domains were detected by using the profile hidden Markov Models for the hmmscan website (https://www.ebi.ac.uk/Tools/hmmer/search/hmmscan). The phylogenetic trees were predicted by the IQ-TREE software (v. 1.6.1) using the Maximum Likelihood with ultrafast bootstrap approach (1000 replicates), and the best-fit model was chosen using ModelFinder incorporated in IQ-TREE [74]. The *TP36/Zator* clade, which is close to *IS630*-*Tc1*-*mariner* group, but forms a separate superfamily [75], was used as an outgroup. Their accession numbers or genome coordinates of *Tc1/mariner* reference elements are listed in Supplementary Table S3. The possible open reading frame (ORF) of *Intruder* identified here and sequences of *RAG1* protein were predicted by GENSCAN website. (http://genes.mit.edu/GENSCAN.html).

### Pairwise distances between IT and RAG1 sequences

Pairwise distances between the various vertebrate organisms used in this study were determined for *IT* and *RAG1* sequences with the purpose of testing the hypothesis of HT by using MEGA7 (highest composite probability and deletion of pairwise) depending on two multiple alignments [76]. The multiple alignments of *IT* consensus sequences derived for each species or representative sequences and *RAG1* coding sequences used to calculate these distances are provided in supplementary files (Text S1 and Text S2), and the access number or genome coordinates of *RAG1*s are listed in Supplementary Table S4.

### Insertion age estimation

To estimate the age of *IT* invasion in each genome, the Kimura two-parameter distance was determined using RepeatMasker’s calcDivergenceFromAlign.pl package from RepeatMasker software [77]. The insertion time of every component was calculated by the eq. T = k/2r [78], where T represents the time of insertion in millions of years, k represents the number of substitutions for nucleotides at each site, and r represents the neutral mutation rates in species lineages. We used the neutral mutation rates for Actinopterygii (1 × 10^− 8^/site/year [79];); Arthropoda (3.46 × 10^− 9^/site/year [80];); *Rhinella marina* (2 × 10^− 9^/site/year [81];); and *Eptatretus burgeri* (1.9 × 10^− 9^/site/year [82];). Because a neutral mutation rate is not available for *Hydra vulgaris*, we used the estimated rate of mutation (5 × 10^− 8^/site/year) for the class Anthozoa [83].

### DNA samples and PCR detection

The blood of all cats used in this experiment was collected from the forelimb vein at the Animal Hospital of Yangzhou University, and then DNA was extracted using Tiangen kit. In order to detect whether *IT* exists in the cat’s genome, we performed a PCR experiment. The PCR was performed with the primer pairs of CAT-FLANK1 under the following conditions: 1 cycle at 95 °C for 5 min; 30 cycles at 95 °C for 30 s, 58 °C for 30 s, 72 °C for 5 min; 1 cycle at 72 °C for 10 min. The PCR was performed with the primer pairs of CAT-FLANK under the following conditions: 1 cycle at 95 °C for 5 min; 30 cycles at 95 °C for 30 s, 58 °C for 30 s, 72 °C for 90s; 1 cycle at 72 °C for 10 min. The PCR was performed with the primer pairs of CAT-CDs under the following conditions: 1 cycle at 95 °C for 5 min; 30 cycles at 95 °C for 30 s, 58 °C for 30 s, 72 °C for 1 min; 1 cycle at 72 °C for 10 min. The PCR was performed with the primer pairs of 5’TIRc, 5’TIRo and 3’TIRc under the following conditions: 1 cycle at 95 °C for 5 min; 30 cycles at 95 °C for 30 s, 58 °C for 30 s, 72 °C for 20s; 1 cycle at 72 °C for 10 min. All primers and pictures are listed in Supplementary Figure S3.

### Gene synthesis and vector construction

To test the transpositioning activity of *IT* in the domesticated “cat” genome, the TIRs and transposase of the intact *IT* copy (AANG04004031.1|:3377–4964) were cloned for a dual (donor and helper) plasmid-based assay. We synthesized the original sequences from the intact copy of cat *IT*, including an ORF of the transposase, the original 5′–terminal repeat sequence (named 5′TIRo) and 3′–terminal repeat sequence (named 3′TIRo). We also obtained the consensus sequence of TIR by alignment, which was 100% identical to 3′TIRo, and 97.62% identical to 5′TIRo. The synthesized ORF sequences of transposases and TIRs are listed in Supplementary Table S5. The 5′TIRo and 3′TIRo sequences were cloned into a pLB vector in turn using restriction enzyme sites. The consensus TIRs (named 5′TIRc and 3′TIRc) were cloned using high fidelity polymerase chain reaction (PCR) amplification from the synthesized 3′TIRo. Then, the neomycin phosphotransferase expression cassette was subcloned from the vector (PB-SB-PGK-Neo-bpA) [36] and inserted between the 5′TIRs and 3′TIRs, and the resulting donor plasmids were named p*IT*o-Neo and p*IT*c-Neo. The synthesized transposase ORF was subcloned into the modified pcDNA3.0 vector with the SV40-Neomycin cassette deleted, and the resulting helper plasmid was named pCMV-itTPase. To construct the two-plasmid system of the *Sleeping Beauty* (*SB*) transposon with the same backbone as *IT*, the neomycin phosphotransferase expression cassette flanked by *SB* TIRs [36] and the SB100X transposase ORF [37] were inserted into the pLB vector and modified pcDNA3.0 vector used by *IT*, respectively. The resulted plasmids named as pSB-Neo and pCMV-SB100X as positive control of transposition activity. The primers for cloning are listed in Supplementary Table S6.

### Cell culture and transposition assays

HeLa and HepG2 cells (American Type Culture Collection, Manassas, VA, USA) were cultured in Dulbecco’s modified Eagle’s medium (DMEM) supplied with 10% foetal bovine serum and 1% penicillin–streptomycin at 37^o^ under 5% CO_2_ in humidified air. For transposition assays, the cells were planted on six-well plates at 3 × 10^5^ /well the day before transfection. 1 μg DNA (donor plasmid and helper plasmid at 1:1 ratio) with 2 μL transfection reagent of Transal-LT1 (Mirus Bio LLC, Madison, WI, USA) was applied for each well. The empty modified vector pcDNA3.0 was used to fill up in negative controls. At 48 h post-transfection, cells were replated on 10 cm dishes and trypsinized (10% or 1% plating). After selection with G418 (1 mg/mL for HeLa cells) for 14 days, cells were 4% paraformaldehyde-fixed, 0.2% methylene blue-stained and blue colonies-counted.

### IT insertion libraries for Sanger sequencing

Following G418 selection (1 mg/mL) for 21 days, HepG2 cells were picked, and genomic DNA was prepared using DNEasy Blood and Tissue kits (Qiagen, Hilden, Germany). Libraries for the integration were built using linker ligation-mediated PCR as described [84]. Briefly, 10 μg aliquots of DNA fragment were digested overnight with DpnI, isolated by 1% agarose gel electrophoresis to extract the largest fragment, and ligated to True-seq linkers after sonication, end repair and dA tailing. Under strict conditions, two rounds of PCR were performed using end-specific primers complementing transposon sequences and linker-specific primers complementing the DNA linker. The second round of PCR products were isolated in 1% agarose gels, and fragments ranging from 200 to 500 bp were recovered using MiniBEST Agarose Gel DNA Extraction kits (TakaRa Bio Inc., Kyoto, Japan) as an insertion library and cloned for Sanger sequencing. Primers applied for insertion libraries are listed in Supplementary Table S7.

## Supplementary Information

**Additional file 1: Fig. S1.** Copy number estimation by BLASTN search against the cat RefSeq genome with the identified *IT* sequence. **Fig. S2.** IT elements (> 80% identity and 40% coverage) were identified in the cat genome. **Fig. S3.** Representative PCR products of IT motifs from the cat genome. (A) Primer sequences for transposon amplification. Nested PCR was used to amplify the transposase gene with two pairs of primers flanking the transposon or matching the CDS. Three pairs of primers were used for TIRs. (B) Schematic diagram of primer locations in the cat genome. These primers were designed using Primer 3. (C) Gel electrophoresis image of PCR products. **Fig. S4.** Alignment of the cat IT sequence to the nucleotide collection (nr/nt) database at NCBI. **Fig. S5.** Full phylogenetic tree of entire IT elements based on the alignment of DDE domains. The phylogenetic tree was inferred using the maximum likelihood method with the IQ-TREE program. Species with incomplete DD38E motifs were excluded from this analysis. **Fig. S6.** Motifs prediction for IT transposases. This analysis was performed using multiple alignment with Bioedit and with modifications in Genedoc. **Fig. S7.** Phylogenetic tree based on the alignment of the nucleotide sequence of IT transposons. The phylogenetic tree was inferred using the maximum likelihood method with the IQ-TREE program based on the alignment of transposon consensus or representative sequences. **Fig. S8.** Insertion ages of ITs. All sequences have consensus or representative sequences. The y-axis represents the mutation rate of each IT element in the genome, and the x-axis represents the age of transposon insertion. This analysis was performed using RepeatMasker. **Fig. S9.** Sequence identity matrix of IT elements. The sequence identities were measured by pairwise comparisons of the transposon consensus sequences or representative sequences. **Fig. S10.** Multiple alignments of the TIRs of copies 1 and 2 with the consensus sequence were performed using Bioedit.

**Additional file 2: Table S1.** Taxonomic distribution of *ITs*.

**Additional file 3: Table S2.** Pairwise distances of *IT* and *RAG1*.

**Additional file 4: Table S3.** The access number of reference *Tc1* elements.

**Additional file 5: Table S4.** The accession numbers of *RAG1*.

**Additional file 6: Table S5.** The synthesised sequences of ORF and TIRs.

**Additional file 7: Table S6.** The primers for *IT* cloning.

**Additional file 8: Table S7.** Primers for insertion libraries.

**Additional file 9.**

**Additional file 10.**

**Additional file 11.**

## Data Availability

All data needed to evaluate the conclusions in this paper are present either in the main text or in the Supplementary Materials.
