# Supplementary material for: Intruder (DD38E), a recently evolved sibling family of DD34E/Tc1 transposons in animals
Source: Mob DNA. 2020 Dec 10;11:32. doi: 10.1186/s13100-020-00227-7 (PMC7731502; doi:10.1186/s13100-020-00227-7)
Supplement: Supplementary file 1 — Additional file 1: Fig. S1. Copy number estimation by BLASTN search against the cat RefSeq genome with the identified IT sequence. Fig. S2. IT elements (> 80% identity and 40% coverage) were identified in the cat genome. Fig. S3. Representative PCR products of IT motifs from the cat genome. (A) Primer sequences for transposon amplification. Nested PCR was used to amplify the transposase gene with two pairs of primers flanking the transposon or matching the CDS. Three pairs of primers were used for TIRs. (B) Schematic diagram of primer locations in the cat genome. These primers were designed using Primer 3. (C) Gel electrophoresis image of PCR products. Fig. S4. Alignment of the cat IT sequence to the nucleotide collection (nr/nt) database at NCBI. Fig. S5. Full phylogenetic tree of entire IT elements based on the alignment of DDE domains. The phylogenetic tree was inferred using the maximum likelihood method with the IQ-TREE program. Species with incomplete DD38E motifs were excluded from this analysis. Fig. S6. Motifs prediction for IT transposases. This analysis was performed using multiple alignment with Bioedit and with modifications in Genedoc. Fig. S7. Phylogenetic tree based on the alignment of the nucleotide sequence of IT transposons. The phylogenetic tree was inferred using the maximum likelihood method with the IQ-TREE program based on the alignment of transposon consensus or representative sequences. Fig. S8. Insertion ages of ITs. All sequences have consensus or representative sequences. The y-axis represents the mutation rate of each IT element in the genome, and the x-axis represents the age of transposon insertion. This analysis was performed using RepeatMasker. Fig. S9. Sequence identity matrix of IT elements. The sequence identities were measured by pairwise comparisons of the transposon consensus sequences or representative sequences. Fig. S10. Multiple alignments of the TIRs of copies 1 and 2 with the consensus sequence were performed using Bioed [file 13100_2020_227_MOESM1_ESM.pdf]

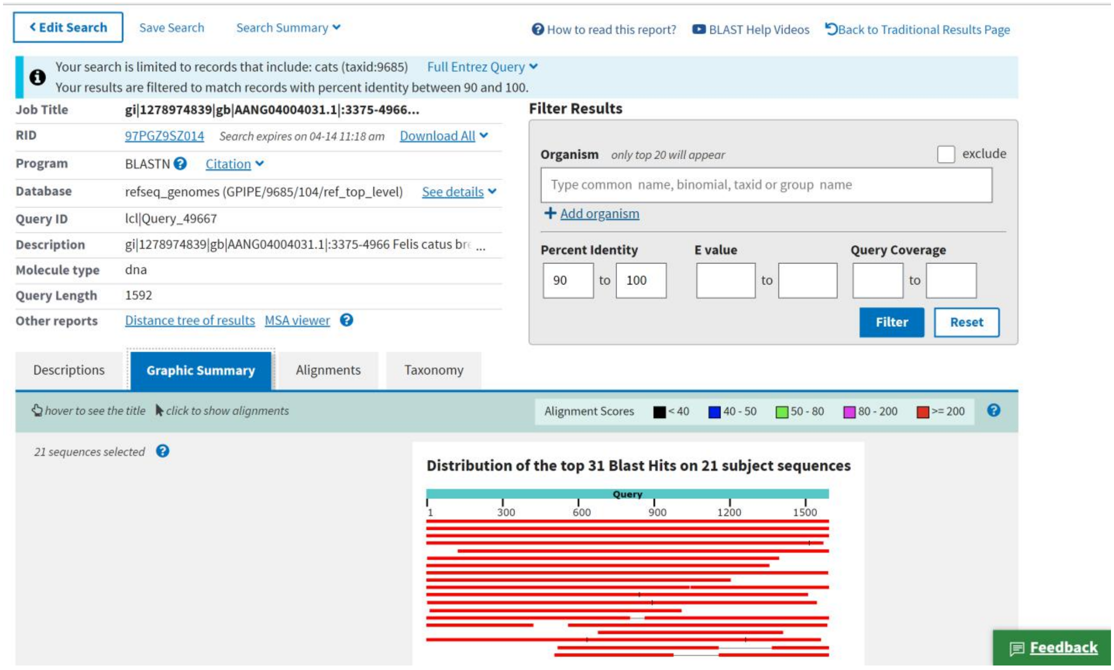

Fig. S1. Copy number estimation by BLASTN search against the cat RefSeq genome with the identified *IT* sequence.

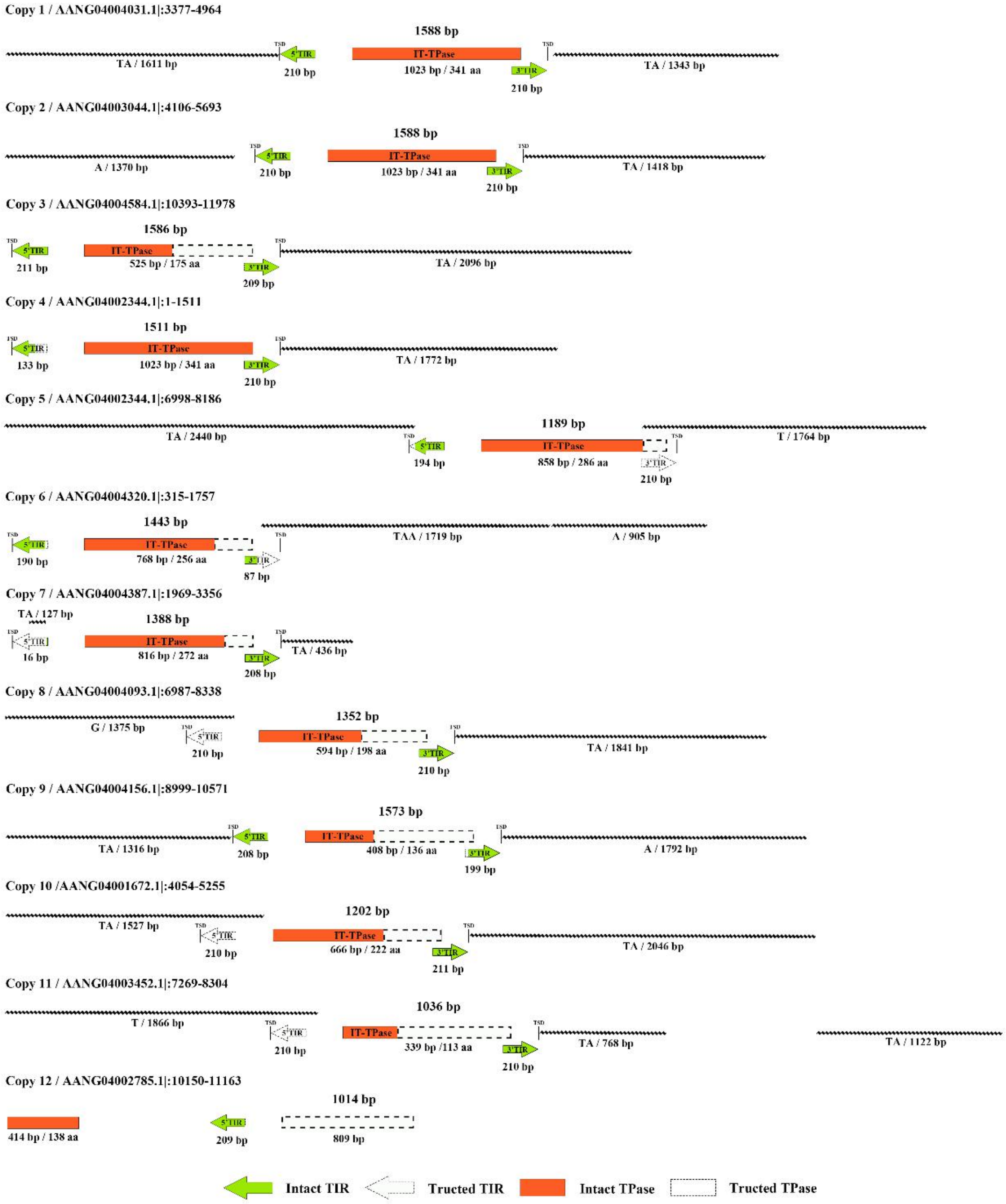

Fig. S2. IT elements (>80% identity and 40% coverage) were identified in the cat genome.

A

CAT-flank1-F: GTTTACACCTGCCCGTCTGT

CAT-flank1-R: AGTGGTGGGGATCATCATGT

CAT-flank-F: ATATATATTATATATATATATATACAGT

CAT-flank-R: TAAAATATTCATTGCTTTTGTTGTAG

CAT-CDS-F: GGATCCGCCACCATGGCAAGACTGAGCACAGC (BamH1)

CAT-CDS-R: GAATTCTCAATATTTGGTGTGACCACCC (EcoR I)

CAT-5'TIRc-F: AGCGCTGGCGCGCCGAGCTC CAGTACTGTGCAAAAGTTTTAG (Afe1, Asc1, Sac1)

CAT-5'TIRc-R: ACTAGTGCTAGC CTACCTGCCGAGTTCCTTCA (Spe1, Nhe1)

CAT-3'TIRc-F: CCGCGGTCGCGA CTACCTGCCGAGTTCCTTCA (SacII, Nru1)

CAT-3'TIRc-R: GGCCGGCCTTAATTAA CAGTACTGTGCAAAAGTTTTAG (Fse1, Pac1)

CAT-5'TIRo-F: AGCGCTGGCGCGCCGAGCTC CAGTACTGTGCAAAAGTTTTAG (Afe1, Asc1, Sac1)

CAT-5'TIRo-R: ACTAGT GCTAGC TGTCTTCAGCAACCTCACCT (Spe1, Nhe1)

B

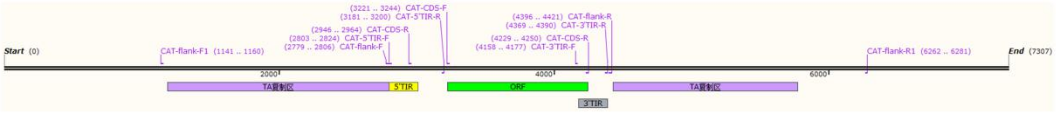

C

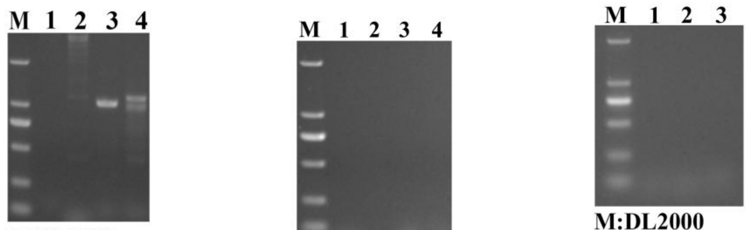

M:DL2000
1、2: CAT-Flank1(5141 bp)
3、4: CAT-CDS (1029 bp)

M:DL2000
1、2: CAT-Flank (1642 bp)
3、4: CAT-CDS (1029 bp)

M:DL2000
1: 5'TIRc (233 bp)
2: 5'TIRo (398 bp)
3: 3'TIRc (233 bp)

Fig. S3. Representative PCR products of IT motifs from the cat genome. (A) Primer sequences for transposon amplification. Nested PCR was used to amplify the transposase gene with two pairs of primers flanking the transposon or matching the CDS. Three pairs of primers were used for TIRs. (B) Schematic diagram of primer locations in the cat genome. These primers were designed using Primer 3. (C) Gel electrophoresis image of PCR products.

select all 100 sequences selected

GenBank Graphics Distance tree of results

	Description	Max Score	Total Score	Query Cover	E value	Per. Ident	Accession
☑	Acipenser transmontanus transposon TC1-like, complete sequence	2898	2898	100%	0.0	99.62%	JX889428.1
☑	Acipenser naccarii transposon TC1-like, complete sequence	2883	2883	100%	0.0	99.43%	JX889427.1
☑	Acipenser nudiventris transposon TC1-like, complete sequence	2856	2856	100%	0.0	99.12%	JX889436.1
☑	Acipenser sturio transposon TC1-like, complete sequence	2839	2839	100%	0.0	98.93%	JX889425.1
☑	Acipenser naccarii transposon TC1-like, complete sequence	2833	2833	100%	0.0	98.87%	JX889426.1
☑	Acipenser stellatus transposon TC1-like, complete sequence	2804	2804	100%	0.0	98.55%	JX889438.1
☑	Acipenser fulvescens transposon TC1-like, complete sequence	2795	2795	100%	0.0	98.37%	JX889435.1
☑	Acipenser mikadoi transposon TC1-like, complete sequence	2795	2795	100%	0.0	98.43%	JX889432.1
☑	Acipenser ruthenus transposon TC1-like, complete sequence	2741	2741	100%	0.0	97.98%	JX889437.1

100 sequences selected

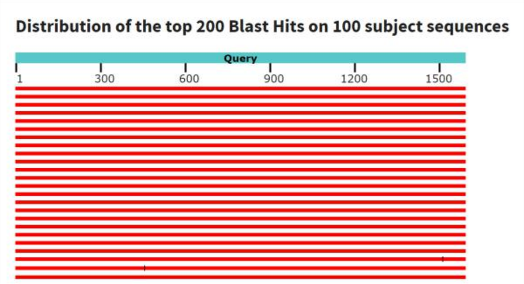

Fig. S4. Alignment of the cat IT sequence to the nucleotide collection (nr/nt) database at NCBI.

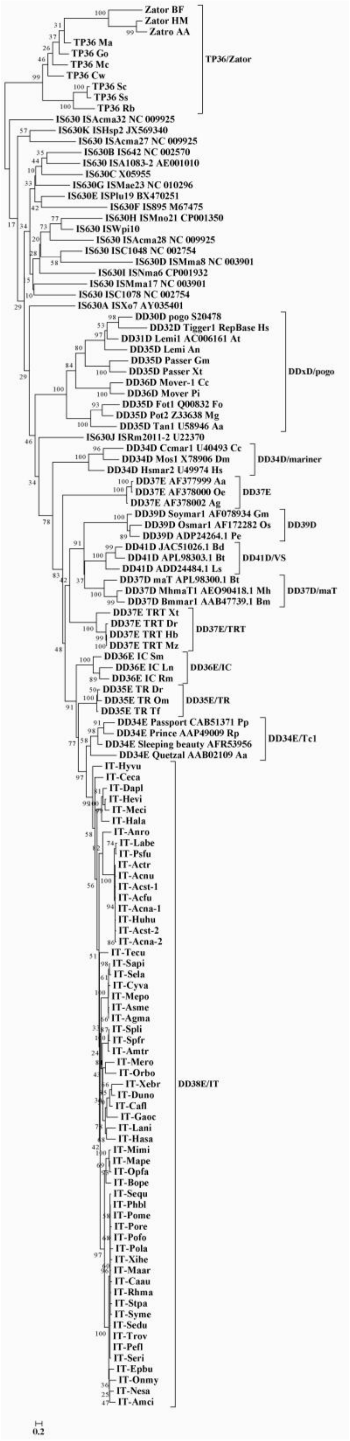

Fig. S5. Full phylogenetic tree of entire IT elements based on the alignment of DDE domains. The phylogenetic tree was inferred using the maximum likelihood method with the IQ-TREE program. Species with incomplete DD38E motifs were excluded from this analysis.

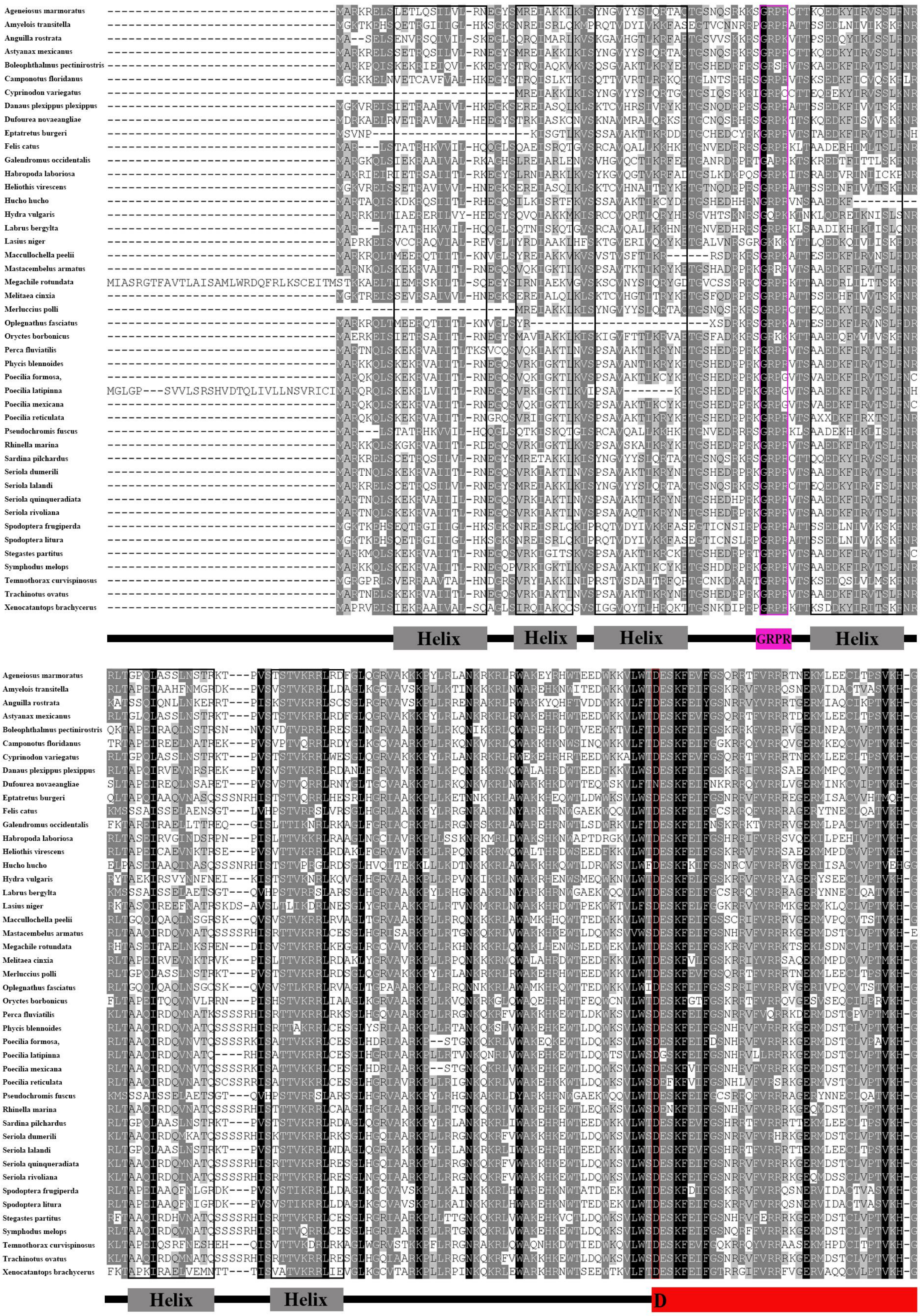

Ageneiosus marmoratus
Amyelois transitella
Anguilla rostrata
Astyanax mexicanus
Boleophthalmus pectinirostris
Camponotus floridanus
Cyprinodon variegatus
Danaus plexippus plexippus
Dufourea novaeangliae
Eptatretus burgeri
Felis catus
Galendromus occidentalis
Habropoda laboriosa
Heliothis virescens
Hucho hucho
Hydra vulgaris
Labrus bergylta
Lasius niger
Macculochella peelii
Mastacembelus armatus
Megachile rotundata
Melitaea cinxia
Merluccius polli
Oplegnathus fasciatus
Oryctes borbonicus
Perca fluviatilis
Phycis blennoides
Poecilia formosa,
Poecilia latipinna
Poecilia mexicana
Poecilia reticulata
Pseudochromis fuscus
Rhinella marina
Sardina pilchardus
Seriola dumerili
Seriola lalandi
Seriola quinqueradiata
Seriola rivoliana
Spodoptera frugiperda
Spodoptera litura
Stegastes partitus
Symphodus melops
Temnothorax curvispinosus
Trachinotus ovatus
Xenocatantops brachycerus
Helix
Helix
Helix
GRPR
Helix
Helix
Helix
D

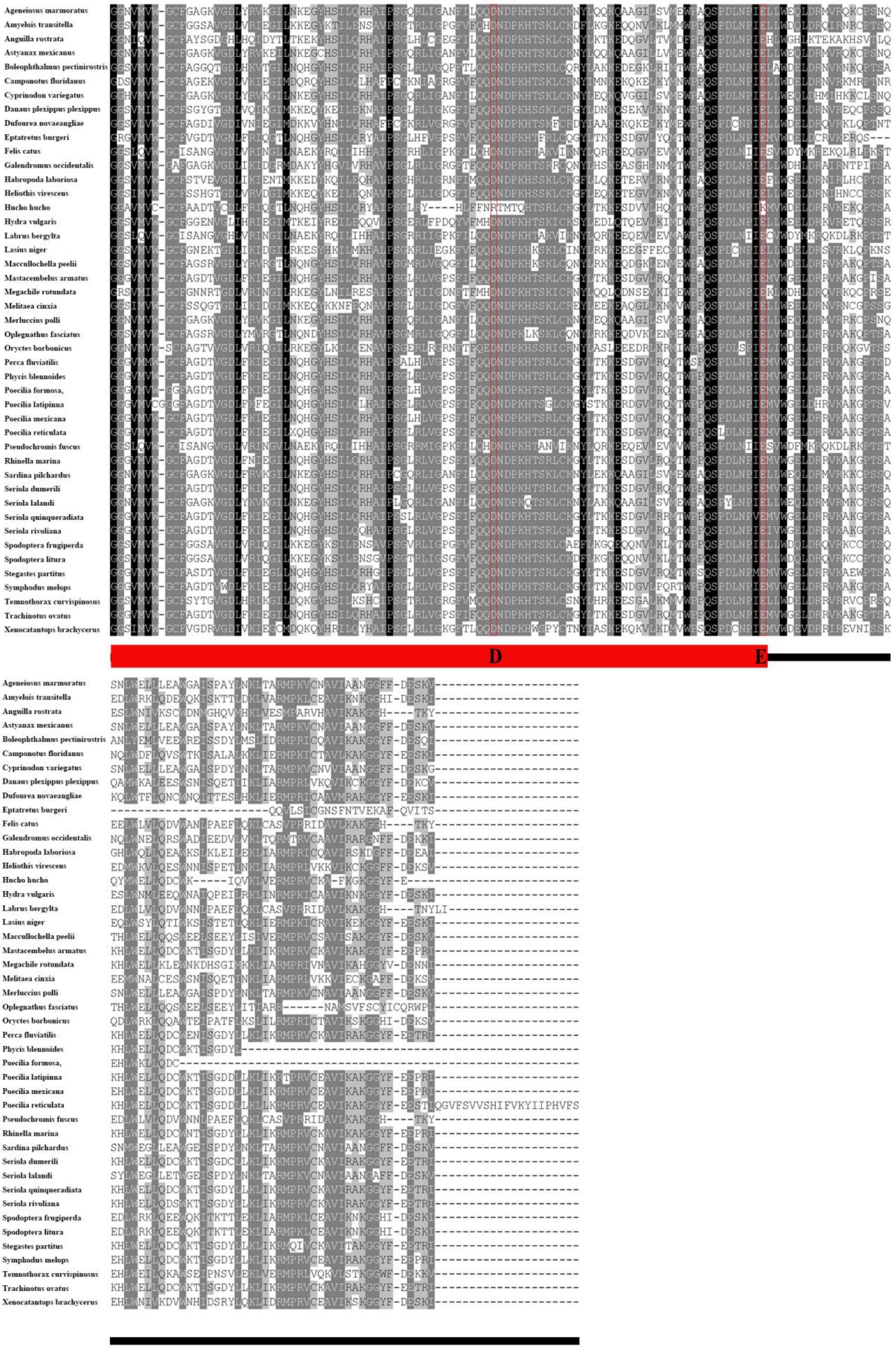

Fig. S6. Motifs prediction for IT transposases. This analysis was performed using multiple alignment with Bioedit and with modifications in Genedoc.

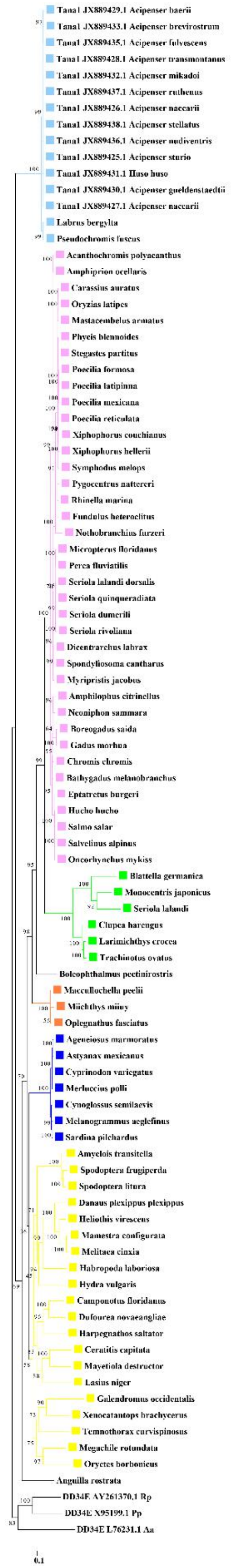

Fig. S7. Phylogenetic tree based on the alignment of the nucleotide sequence of IT transposons. The phylogenetic tree was inferred using the maximum likelihood method with the IQ-TREE program based on the alignment of transposon consensus or representative

sequences.

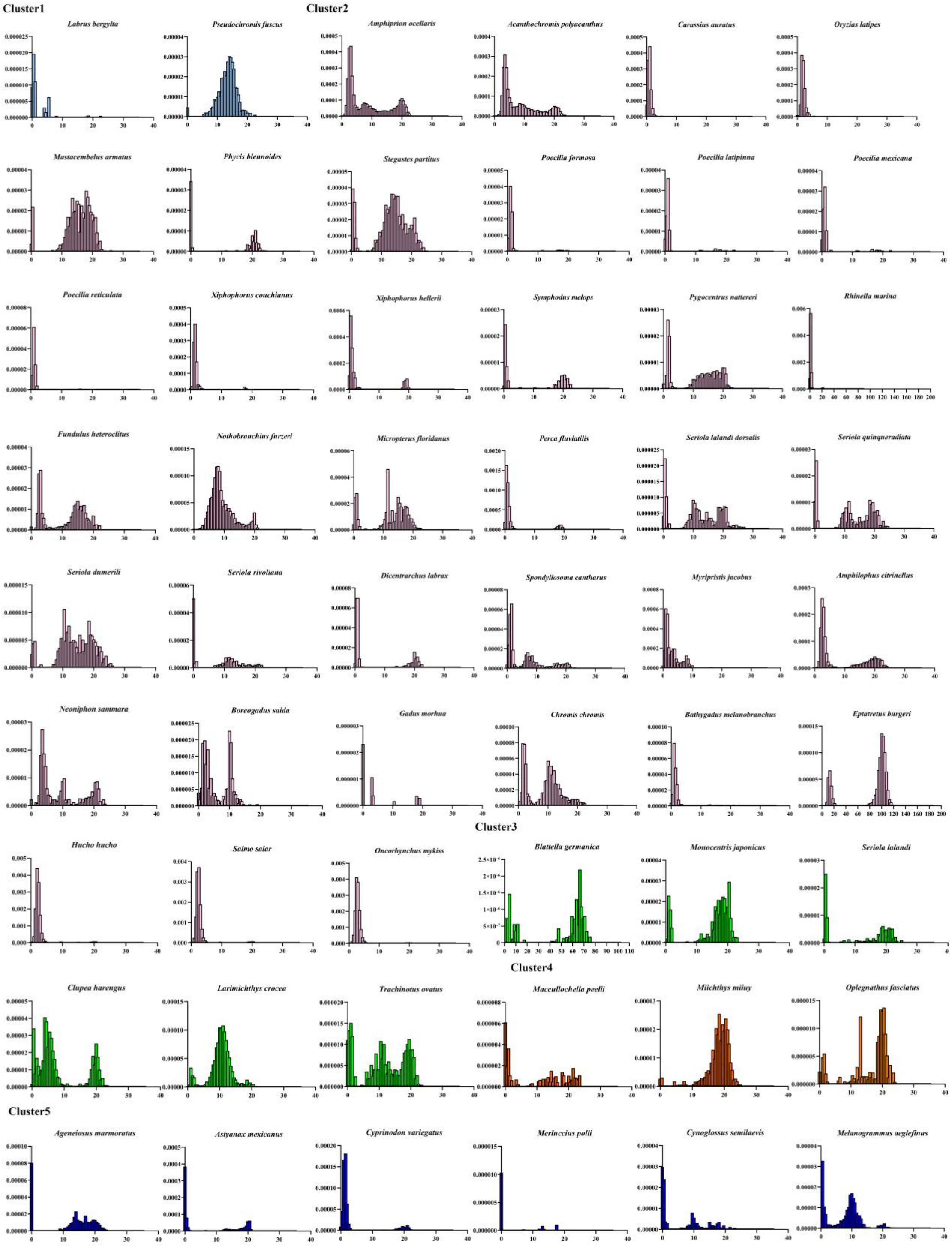

Cluster1
Labrus bergylta
Pseudochromis fuscus
Cluster2
Amphiprion ocellaris
Acanthochromis polyacanthus
Carassius auratus
Oryzias latipes
Mastacembelus armatus
Phycis blennoides
Stegastes partitus
Poecilia formosa
Poecilia latipinna
Poecilia mexicana
Poecilia reticulata
Xiphophorus couchianus
Xiphophorus hellerii
Symphodus melops
Pygocentrus nattereri
Rhinella marina
Fundulus heteroclitus
Nothobranchius furzeri
Micropterus floridanus
Perca fluviatilis
Seriola lalandi dorsalis
Seriola quinqueradiata
Seriola dumerili
Seriola rivoliana
Dicentrarchus labrax
Spondyliosoma cantharus
Myripristis jacobus
Amphilophus citrinellus
Neoniphon sammara
Boreogadus saida
Gadus morhua
Chromis chromis
Bathygadus melanobranchus
Eptatretus burgeri
Cluster3
Hucho hucho
Salmo salar
Oncorhynchus mykiss
Blattella germanica
Monocentris japonicus
Seriola lalandi
Cluster4
Clupea harengus
Larimichthys crocea
Trachinotus ovatus
Maccullochella peelii
Miichthys miiuy
Oplegnathus fasciatus
Cluster5
Ageneiosus marmoratus
Astyanax mexicanus
Cyprinodon variegatus
Merluccius polli
Cynoglossus semilaevis
Melanogrammus aeglefinus

Fig. S8. Insertion ages of ITs. All sequences have consensus or representative sequences. The y-axis represents the mutation rate of each IT element in the genome, and the x-axis represents the age of transposon insertion. This analysis was performed using RepeatMasker.

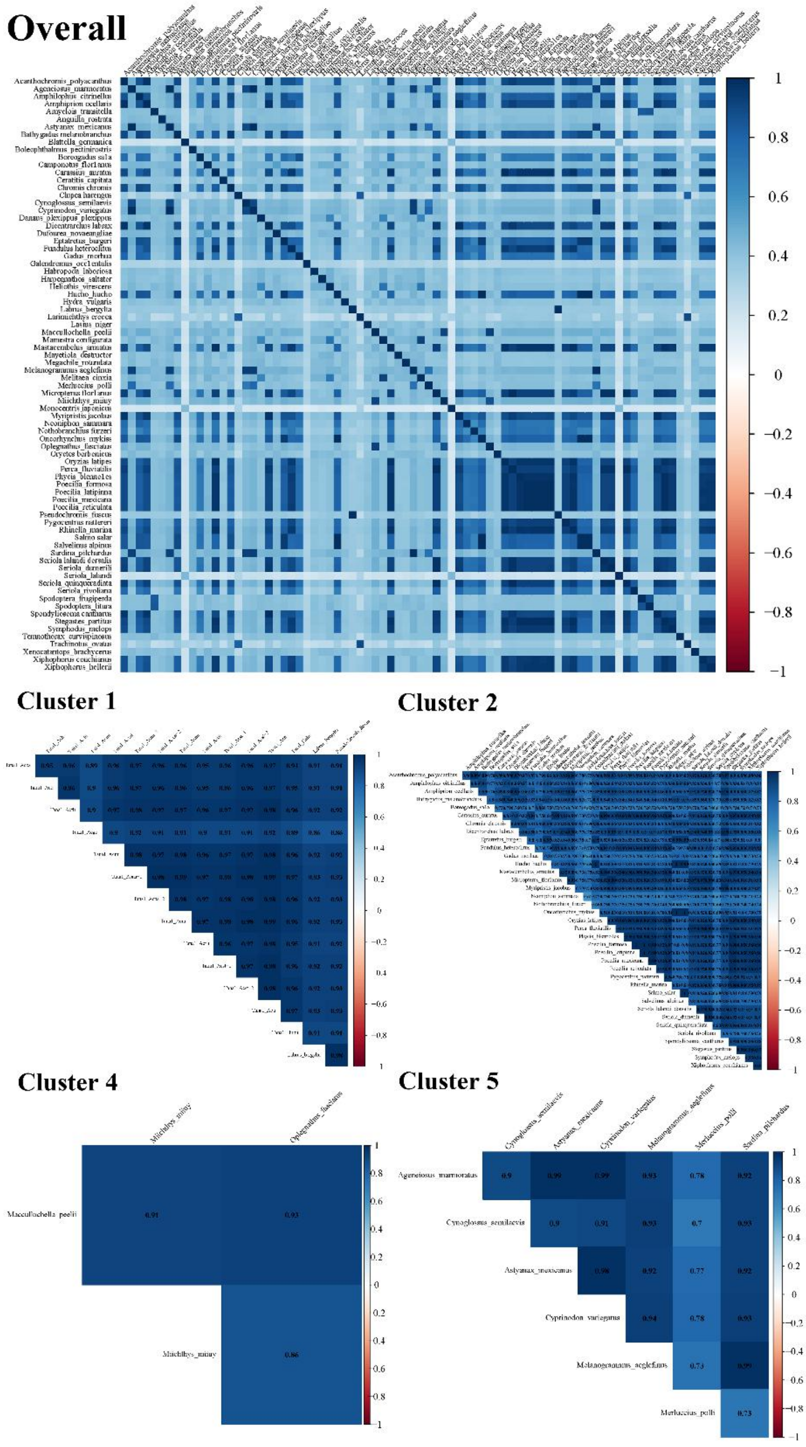

Fig. S9. Sequence identity matrix of IT elements. The sequence identities were measured by pairwise comparisons of the transposon consensus sequences or representative sequences.

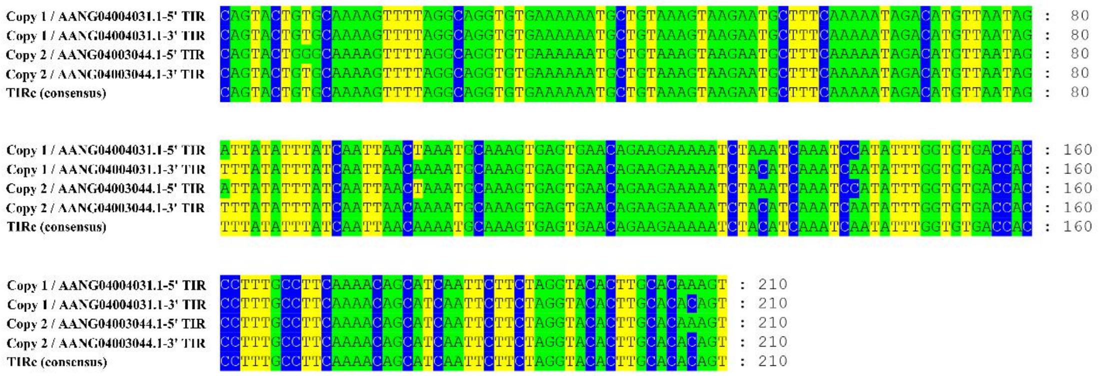

Fig. S10. Multiple alignments of the TIRs of copies 1 and 2 with the consensus sequence were performed using Bioedit.
