# Supplementary material for: Intruder (DD38E), a recently evolved sibling family of DD34E/Tc1 transposons in animals
Source: Mob DNA. 2020 Dec 10;11:32. doi: 10.1186/s13100-020-00227-7 (PMC7731502; doi:10.1186/s13100-020-00227-7)
Supplement: Supplementary file 6 — Additional file 6: Table S5. The synthesised sequences of ORF and TIRs. [file 13100_2020_227_MOESM6_ESM.docx]

| itTp |
| --- |
| >felis catus Positions 425 to 1447 |
| GGATCCGCCACCATGGCAAGACTGAGCACAGCAACAAGACACAAGGTAGTTATACTGCATCAGCAAGGTCTCTCCCAGGCAGAAATTTCAAGGCAGACAGGGGTTTCCAGATGTGCTGTCCAAGCTCTTTTGAAGAAGCACAAAGAAACGGGCAACGTTGAGGACCGTAGACGCAGTGGTCGGCCAAGGAAACTTACTGCAGCAGATGAAAGACACATCATGCTTACTTCCCTTCGCAATCGGAAGATGTCCAGCAGTGCCATCAGCTCAGAATTGGCAGAAAACAGTGGGACCCTGGTACACCCATCTACTGTCCGGAGAAGTCTGGTCAGAAGTGGCCTTCATGGAAGACTTGCGGCCAAAAAGCCATACCTCCGACGTGGAAACAAGGCCAAGCGACTCAACTATGCACGAAAACACAGGAACTGGGGTGCAGAAAAATGGCAGCAGGTGCTCTGGACTGATGAGTCAAAATTTGAAATATTTGGCTGTAGCAGAAGGCAGTTTGTTCGCCGAAGGGCTGGAGAGCGGTACACGAATGAGTGTCTGCAGGCAACAGTGAAGCATGGTGGAGGTTCCTTGCAAGTTTGGGGCTGCATTTCTGCAAATGGAGTTGGGGATTTGGTCAGAATTAATGGTCTCCTCAATGCTGAGAAGTACAGGCAGATACTTATCCATCATGCAATACCATCAGGGAGGCATCTGATTGGCCCCAAATTTATTCTGCAGCATGACAACGACCCCAAACATACAGCGAAAGTCATTAAGAACTATCTTCAGCGTAAAGAAGAACAAGGAGTCCTGGAAGTGATGGTATGGCCCCCACAGAGCCCTGATCTCAACATCATCGAGTCTGTCTGGGATTACATGAAGAGAGAGAAGCAACTGAGGCTGCCTAAATCCACAGAAGAACTGTGGTTAGTTCTCCAAGATGTTTGGGCCAACCTACCTGCCGAGTTCCTTCAAAAACTGTGTGCAAGTGTACCTAGAAGAATTGATGCTGTTTTGAAGGCAAAGGGTGGTCACACCAAATATTGAgaattc |
| 5‘TIRo |
| >felis catus Positions 1 to 212 |
| AGCGCTggcgcgccGAGCTCCAGTACTGTGCAAAAGTTTTAGGCAGGTGTGAAAAAATGCTGTAAAGTAAGAATGCTTTCAAAAATAGACATGTTAATAGATTATATTTATCAATTAACTAAATGCAAAGTGAGTGAACAGAAGAAAAATCTAAATCAAATCCATATTTGGTGTGACCACCCTTTGCCTTCAAAACAGCATCAATTCTTCTAGGTACACTTGCACAAAGTgctagcACTAGT |
| 3‘TIRc |
| > felis catus Positions 1 to 212 |
| CCGCGGtcgcgaACTGTGTGCAAGTGTACCTAGAAGAATTGATGCTGTTTTGAAGGCAAAGGGTGGTCACACCAAATATTGATTTGATGTAGATTTTTCTTCTGTTCACTCACTTTGCATTTTGTTAATTGATAAATATAAACTATTAACATGTCTATTTTTGAAAGCATTCTTACTTTACAGCATTTTTTCACACCTGCCTAAAACTTTTGCACAGTACTGttaattaaGGCCGGCC |

Table S5 The synthesised sequences of open reading frame (ORF) of transposase and TIRs
